# The Folding of the Specific DNA Recognition Subdomain of the *Sleeping Beauty* Transposase Is Temperature-Dependent and Is Required for Its Binding to the Transposon DNA

**DOI:** 10.1371/journal.pone.0112114

**Published:** 2014-11-06

**Authors:** Gage O. Leighton, Tatiana A. Konnova, Bulat Idiyatullin, Sophia H. Hurr, Yuriy F. Zuev, Irina V. Nesmelova

**Affiliations:** 1 Department of Physics and Optical Science, University of North Carolina, Charlotte, North Carolina, United States of America; 2 Kazan Institute of Biochemistry and Biophysics, Kazan, Russian Federation; 3 Center for Biomedical Engineering and Science, University of North Carolina, Charlotte, North Carolina, United States of America; Friedrich-Loeffler-Institute, Germany

## Abstract

The reaction of DNA transposition begins when the transposase enzyme binds to the transposon DNA. Sleeping Beauty is a member of the mariner family of DNA transposons. Although it is an important tool in genetic applications and has been adapted for human gene therapy, its molecular mechanism remains obscure. Here, we show that only the folded conformation of the specific DNA recognition subdomain of the Sleeping Beauty transposase, the PAI subdomain, binds to the transposon DNA. Furthermore, we show that the PAI subdomain is well folded at low temperatures, but the presence of unfolded conformation gradually increases at temperatures above 15°C, suggesting that the choice of temperature may be important for the optimal transposase activity. Overall, the results provide a molecular-level insight into the DNA recognition by the Sleeping Beauty transposase.

## Introduction

DNA transposons are mobile genetic elements that can move (transpose) from one location to another in the host genome. Because of this ability, they can be used for gene delivery to vertebrate organisms and their cells [1]–[4]. A typical DNA transposon gene delivery system consists of two components: a transposon DNA containing the gene of interest flanked by inverted terminal repeats (**IR**s) and a transposase enzyme that catalyzes gene transfer. Several DNA transposons are currently being developed and used for genetic applications, including piggyBac, Tol2, Frog Prince, and Sleeping Beauty (**SB**) transposons [5]. SB transposon was the first transposon capable of gene transfer in vertebrate cells [6]. Moreover, the SB transposon is the first and only DNA transposon that, currently, is in a clinical trial for human gene therapy [4]. Therefore, understanding the molecular mechanisms of SB transposition is of great interest.

The SB transposase is a modular protein that contains two functionally and structurally independent domains, the DNA-binding and the catalytic domains. The DNA-binding domain further consists of two structurally independent subdomains, the PAI and the RED subdomains [6]. The PAI subdomain is the primary DNA-recognition subdomain of SB transposase [7]–[8]. It forms a compact, three-helix structure, in which helices 2 and 3 form a helix-turn-helix DNA-binding motif [7].

The efficiency of the DNA transposon delivery system depends on a variety of factors, including physical and biochemical conditions such as the concentration of the transposase enzyme (i.e., overproduction inhibition, recently reviewed by Bire et al. [9]), pH [10], or temperature [11]–[12]. In general, examples of the temperature-dependent transposition have been reported for a large number of transposable elements of diverse origin and utilizing different mechanisms [13]–[20]. The dependence of transposition activity on temperature could be due to a number of reasons, such as the production of truncated transposases [19], the increase of auto-integration events [12], the difference in subcellular localizations of the transposase [13], levels of transposase expression [20], or its DNA binding properties [13].

The present study highlights yet another cause that may affect the activity of transposase enzyme during the reaction of transposition, namely the temperature-induced structural change due to unfolding of the whole protein or some regions within the protein. Using Nuclear Magnetic Resonance (**NMR**) spectroscopy and intrinsic tyrosine fluorescence, we show that the content of unfolded state of the primary DNA-recognition subdomain of SB transposase, e.g. the PAI subdomain, gradually increases and becomes noticeable at temperatures above 15°C at physiologic pH. We further show that the transposon DNA preferentially binds the folded conformation of the PAI subdomain, suggesting that the temperature-induced unfolding may affect the SB transposase DNA-binding properties, and hence its activity. Altogether, the results provide a molecular-level insight into the mechanism of the transposon DNA recognition by the PAI subdomain of SB transposase.

## Materials and Methods

### Protein expression, purification and sample preparation

The PAI subdomain was expressed and purified using established protocol that we have previously reported [7]. DNA plasmid coding the His-tagged PAI subdomain of SB transposase (N terminal residues G1-Q53), cloned into pET 21a(+) vector, was ordered from GenScript USA Inc. (Piscataway, NJ, USA). Recombinant protein was expressed in a soluble form in BL21-A1 *E. coli* cells and purified using Ni-affinity chromatography. ^15^N-labeled (for 2D NMR spectroscopy) or unlabeled (for self-diffusion, fluorescence, and light scattering experiments) protein samples were prepared in 25 mM sodium phosphate buffer at pH 5.0 or 7.0. 10%/90% D_2_O/H_2_O was used to prepare NMR samples for 2D [^1^H,^15^N]-HSQC experiments. 100% D_2_O was used in samples prepared for self-diffusion measurements.

### NMR spectroscopy

All 2D NMR experiments were carried out on a Bruker Avance-III 700 or 950 MHz spectrometers equipped with CryoProbe. Previously reported NMR chemical shift assignments [7] were used. 2D [^1^H,^15^N]-HSQC (heteronuclear single quantum coherence) spectra were used to monitor structural changes or DNA binding. The NMR data were processed with the NMRpipe program [21] and visualized using the NMRView and CARA programs [22]–[23].

Self-diffusion coefficients, *D*, were measured by pulsed-field gradient (**PFG**) NMR on a Bruker Avance-III 600 MHz spectrometer equipped with a z-gradient inverse detection probe. The maximum magnitude of the pulsed-filed gradient, *g*, was calibrated using deuterated water standard and was equal to 55.7 G cm^−1^. The experiments were performed using a stimulated-echo sequence incorporating bipolar gradient pulses and a longitudinal eddy current delay (**BPP-LED**) [24]. Water suppression was achieved by presaturation. The value of the self-diffusion coefficient was estimated from the diffusion attenuation of spin echo amplitude, *A*(*g* ^2^)  =  *A*(0)·exp(-*γ^2^δ^2^g^2^Dt_d_*), where *γ* is the gyromagnetic ratio for protons, *δ* is the duration of the PFG, and *t_d_* is the diffusion time, which comprises all time delays between pulses, during which the magnetization is oriented along the z-axis [25].

### Intrinsic tyrosine fluorescence and Rayleigh Light Scattering

Intrinsic tyrosine fluorescence and right-angle static light scattering (**SLS**) measurements were done on a PTI (Photon Technology International) QuantaMaster fluorescence spectrofluorometer on the same sample. The temperature of the jacketed cell-holder was maintained by circulating water and monitored by a Hanna Instruments 93530 K-thermocouple thermometer. The excitation wavelength was 275 nm, and the fluorescence emission was collected from 290 to 450 nm. Fluorescence data are presented as the total integral fluorescence intensity, IF, calculated by integrating the area under the fluorescence curve. IF is temperature-dependent and decreases with increasing temperature according to the Arrhenius law, i.e ∼ exp (- *E_eff_*/*RT*), where R is the gas constant and *E_eff_* can be interpreted as the activation energy of the processes that lead to fluorescence quenching [26]–[27]. Temperature dependence was ranged from 5°C to 55°C at both pH 5.0 and 7.0. The reported data is the average of three independent measurements of the temperature dependence of IF done using a new protein sample.

## Results

**Figure 1:**
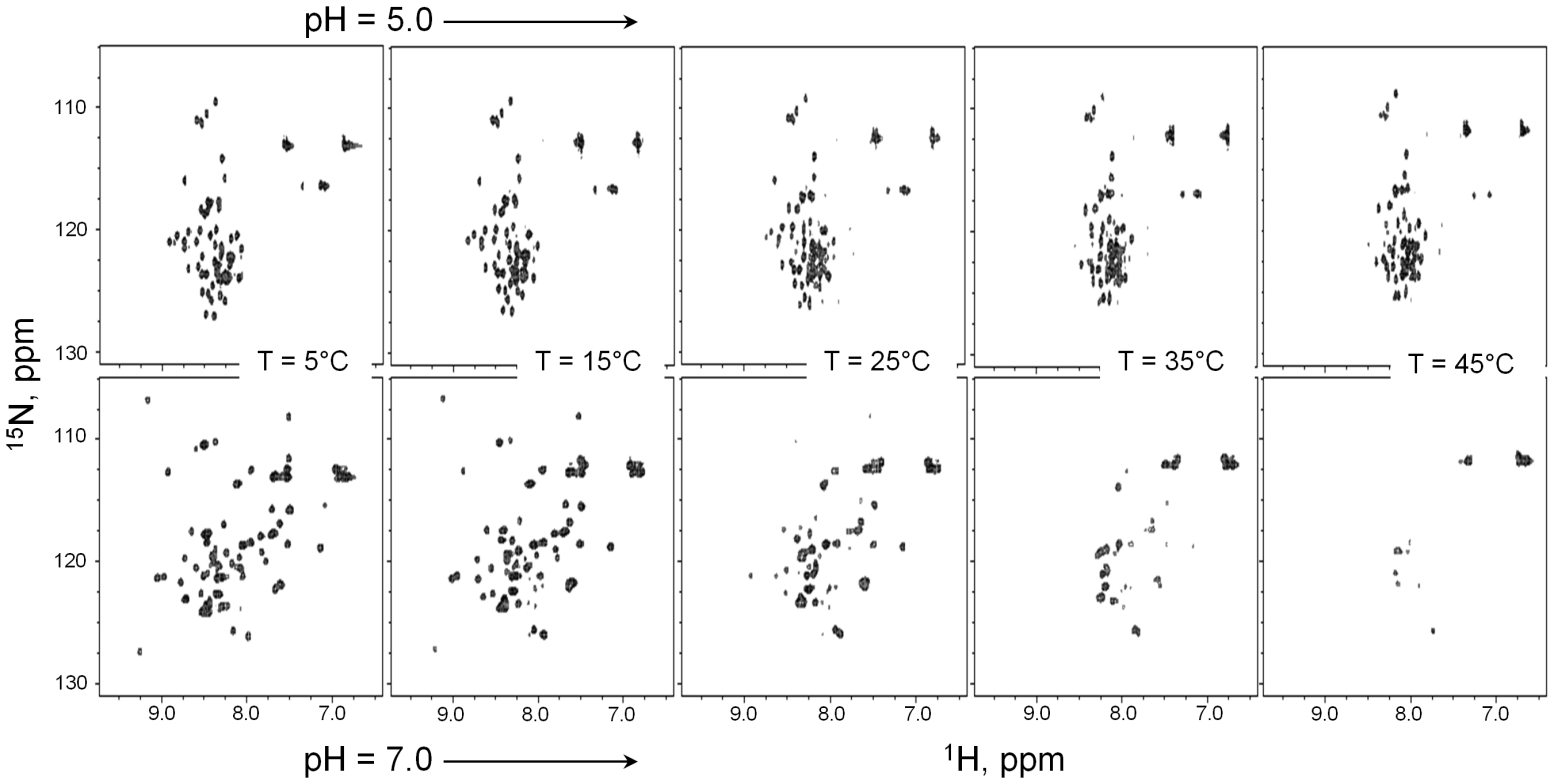
2D [^1^H,^15^N]-HSQC spectra of the PAI subdomain. The spectra were collected in 25 mM aqueous sodium phosphate buffer at pH 5.0 (top panel) and 7.0 (bottom panel) in the range of temperatures from 5 to 45°C with a 5°C increment.

Previously, we have shown that folding properties of the PAI subdomain of SB transposase depend on solution pH [7]. Figure 1 shows a series of 2D [^1^H,^15^N]-HSQC spectra of the PAI subdomain collected at solution pH values 5.0 and 7.0 and at temperatures varying from 5 to 45°C. The [^1^H,^15^N]-HSQC at 5°C and pH 5.0 reveals very limited chemical shift dispersion, with the majority of the ^1^H resonances around 8 ppm. This indicates that the respective amino acid residues are in random coil conformation and the PAI subdomain is essentially unfolded. In contrast, at pH 7.0, many resonances shift and become well dispersed, indicative of a folded structure. Increasing the temperature has very little effect on the chemical shift dispersion and signal intensity in the [^1^H,^15^N]-HSQC spectrum of the PAI subdomain at pH 5.0, implying that the PAI subdomain remains unfolded at all temperatures between 5 and 45°C. On the contrary, drastic changes are observed in the [^1^H,^15^N]-HSQC spectra of the PAI subdomain at pH 7.0. As the temperature increases, many signals become severely broadened and only a few signals remain observable at temperatures above 35°C. Observed signal broadening may be due to the conformational exchange between the folded and unfolded states of the protein, protein aggregation, or both.

**Figure 2:**
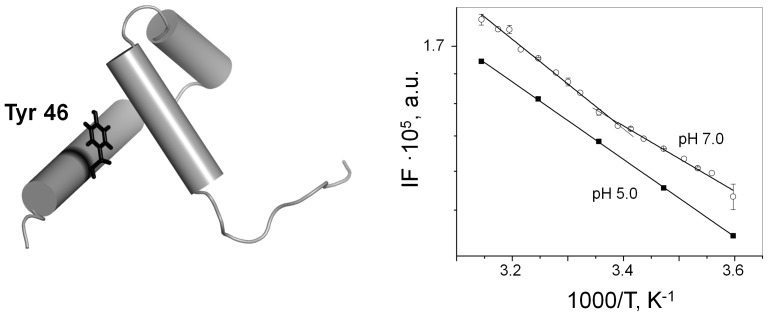
Intrinsic tyrosine fluorescence. (Left panel) The location of Y46 on the cartoon representation of the PAI subdomain (PDB code 2m8e [7]) is shown. (Right panel) The integral fluorescence of Y46 at pH 5.0 (squares) and pH 7.0 (circles) is plotted vs. temperature. Shown data is the average of three independent experiments. Error bars in many cases do not exceed the size of the symbol. Solid lines represent best fits of experimental data.

Intrinsic tyrosine fluorescence was used to monitor the change of the PAI subdomain folding state with temperature. Proteins derive their intrinsic fluorescence from the chromophores phenylalanine, tyrosine, and tryptophan. The PAI subdomain contains only one tyrosine (Y46) and does not have any phenylalanine or tryptophan residues. The side chain of Y46 is oriented towards the interior of the protein (Figure 2, left panel), and thus should be sensitive to the PAI subdomain unfolding. The total integral fluorescence intensity as a function of temperature is plotted in Figure 2 (right panel) for pH values of 5.0 and 7.0 using a semi-logarithmic scale. At pH 5.0, the temperature dependence of Y46 fluorescence is linear, as expected, because the PAI subdomain remains unfolded and the environment of Y46 does not change. At pH 7.0, the transition between 18 and 28°C is observed, indicating that there is a change in the Y46 environment due to PAI unfolding, in agreement with our [^1^H,^15^N]-HSQC data shown in Figure 1. We note that the effect is not very strong. This is likely due to the fact that even in a folded state Y46 has good water accessibility due to the small size and flexibility of the PAI subdomain.

**Figure 3:**
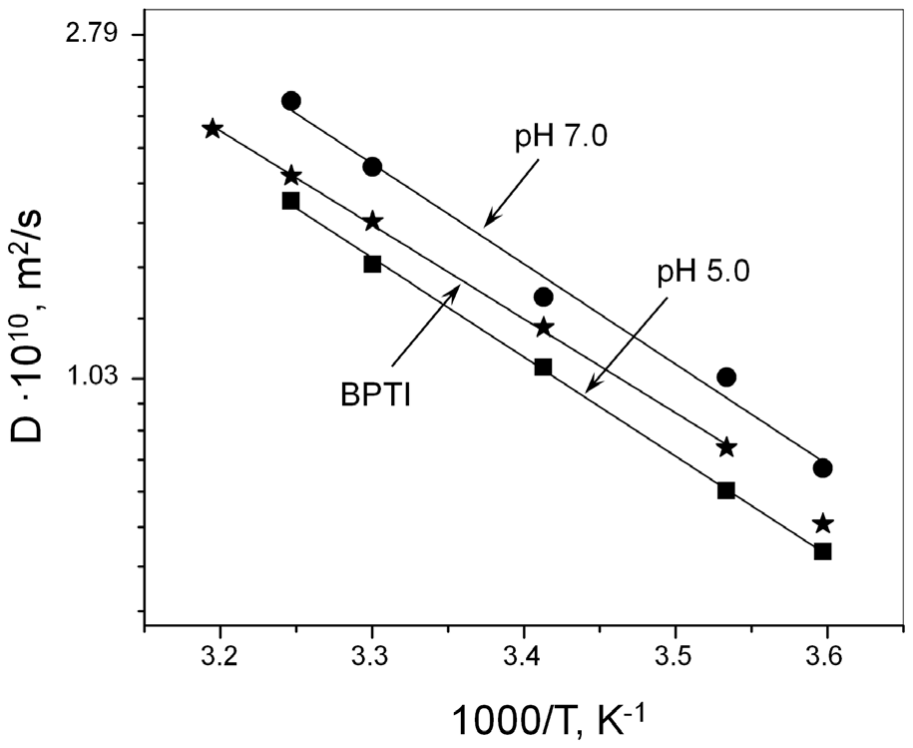
Self-diffusion coefficients. The PAI self-diffusion coefficient at pH 5.0 (squares) and 7.0 (circles) are plotted vs. temperature over the range from 5 to 35°C. Protein samples were prepared in 25 mM sodium phosphate buffer using 100% D_2_O. The temperature dependence of the self-diffusion coefficient of BPTI (stars) is shown for comparison. Solid lines represent fits of Arrhenius dependence of the self-diffusion coefficient to experimental data.

According to the Stokes-Einstein equation *D =  k_B_T/6*πη*R*, the self-diffusion coefficient is inversely proportional to the radius, *R*, of the diffusing species in solution; hence, it was used to determine whether the aggregation state of the PAI subdomain changes with temperature. Other quantities in the Stokes-Einstein equation include the Boltzmann constant *k_B_* and the viscosity of pure solvent η (e.g., D_2_O). In the absence of processes that could lead to the change of protein size with temperature, i.e. protein aggregation or unfolding, the temperature dependence of *D* is determined only by the temperature dependence of the viscosity η of D_2_O. Accordingly, it is expected to follow the Arrhenius relation with a slope reflecting the activation energy of the self-diffusion of water (5 kcal/mol) [28]. Figure 3 shows the temperature dependence of the PAI self-diffusion coefficient at pH 5.0 (squares) and 7.0 (circles) over the temperature range 5–35°C. The temperature dependence of the self-diffusion coefficient of bovine pancreatic trypsin inhibitor (**BPTI**), which has a comparable molecular weight (6.5 kDa for BPTI vs. 6.9 kDa for PAI) and remains monomeric and folded [29] in the temperature range from 10 to 42°C, was also measured and is shown in Figure 3 for comparison (stars). Solid lines represent fits of Arrhenius dependence of the self-diffusion coefficient to experimental data. The fit for BPTI was done in the interval of temperatures corresponding to its monomeric state. Several conclusions are apparent from Figure 3. (1) The self-diffusion coefficients of the PAI subdomain measured at pH 5.0 and pH 7.0 are different at all temperatures. (2) The temperature dependence of PAI self-diffusion coefficient is linear throughout the entire temperature range, with the slope corresponding to the activation energy of 5.7 kcal/mol at pH 7.0 and 5.8 kcal/mol at pH 5.0. (3) The self-diffusion coefficient of the PAI subdomain is close to the self-diffusion coefficient of BPTI by magnitude, with the self-diffusion coefficient somewhat lower and higher than that of BPTI at pH 5.0 and 7.0, respectively. (4) The slopes of the temperature dependence of PAI self-diffusion coefficient are slightly steeper than the slope of the BPTI self-diffusion coefficient (5.4 kcal/mol) at both pH values.

The following picture emerges based on these observations. The PAI subdomain remains monomeric at all temperatures based on similar values of self-diffusion coefficients observed for the PAI subdomain and BPTI and the linearity of the temperature dependence of the PAI self-diffusion coefficient. In addition, to rule out the formation of protein aggregates, we carried out light scattering experiments and confirmed that the Rayleigh factor at scattering angle 90^o^ remains constant over the whole temperature range 5–45°C (data not shown). This was corroborated by the fact that while the line broadening was observed in 1D NMR diffusion spectra, no significant decrease of the integral spin-echo intensity, which would indicate the loss of contribution from protein aggregates with short T2 relaxation times, was observed. At pH 5.0, the PAI subdomain remains unfolded at all temperatures, in agreement with [^1^H,^15^N]-HSQC data (Figure 1). Thus, it is more likely to behave as a flexible polymer chain rather than the rigid sphere as assumed by the Stokes-Einstein equation, and the activation energy of the PAI self-diffusion is higher than that of water [30]. At pH 7.0, the PAI subdomain is folded at 5°C, in agreement with the observed chemical shift dispersion in the [^1^H,^15^N]-HSQC spectra. The self-diffusion coefficient of a more compact, folded PAI subdomain is approximately 80% larger than the self-diffusion coefficient of unfolded PAI subdomain at pH 5.0. Such an increase of self-diffusion coefficient due to protein unfolding is not unusual and is well within the range of reported values from 38% for lysozyme [31] or 75% for a 130-residue fragment (D1–D4) of a fibronectin-binding protein [32] to 2-fold difference for p53 [33]. The observed difference in self-diffusion coefficients of BPTI and folded PAI subdomain is likely due to the difference in protein concentrations [29], [34], which were 10 and 1.8 mg/mL respectively. As temperature increases, the PAI subdomain undergoes gradual unfolding and consists as an interconverting conformational ensemble, resulting in resonance broadening. The effect becomes noticeable in the [^1^H,^15^N]-HSQC spectra above 15°C (Figure 1). Accordingly, due to the contribution of the unfolded PAI subdomain, the slope of the temperature dependence of *D* is larger for the PAI subdomain than for BPTI, because the measured self-diffusion coefficient is a weighted average over folded and unfolded protein species diffusing in solution.

**Figure 4:**
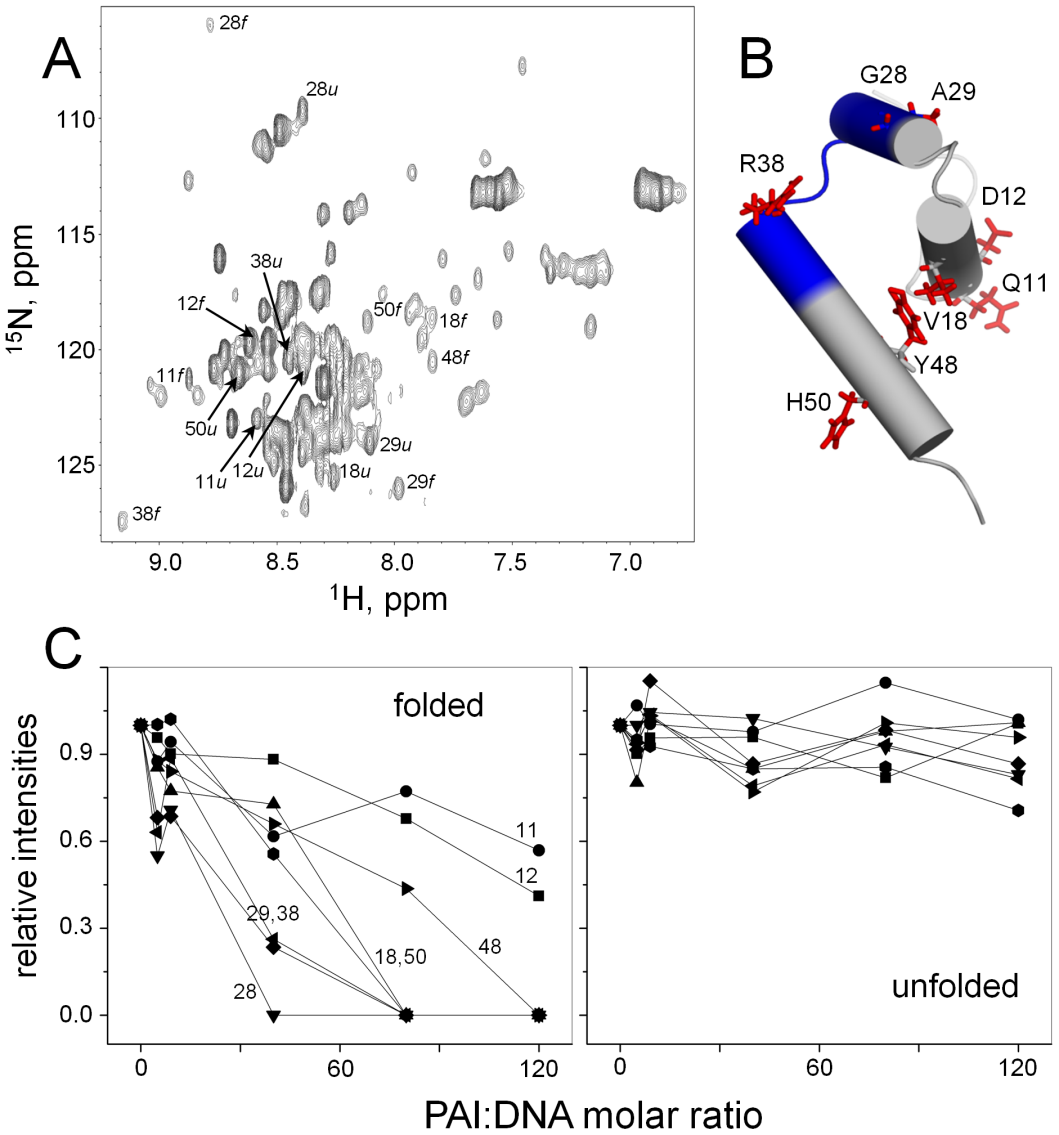
DNA-binding of folded and unfolded conformations of the PAI subdomain. (A) 2D [^1^H,^15^N]-HSQC spectrum of the PAI subdomain collected at the temperature of 5°C in 25 mM aqueous sodium phosphate buffer at pH 5.0 in the presence of 250 mM NaCl. The folded and unfolded conformations exist in slow exchange on the NMR time scale. Thus, two resonances are observed for each residue. Non-overlapping resonances originating from the same residue in both the folded and unfolded conformations are labeled. (B) Cartoon representation of the PAI subdomain (PDB code 2m8e [7]). The DNA-binding site is colored blue. Side chains of the residues that were used for the analysis of the DNA-binding are labeled and shown as red sticks. (C) Relative intensities of resonances corresponding to the folded and unfolded conformations are plotted as a function of PAI:DNA molar ratio. Relative intensities were calculated by dividing the resonance intensity at a given PAI:DNA molar ratio by the intensity of this resonance in the absence of DNA.

The PAI subdomain is the primary DNA-recognition subdomain of SB transposase. We were interested to determine whether the folding of the PAI subdomain is required for binding the transposon DNA, because it could have direct implications for the function of SB transposase. The PAI subdomain exists in the equilibrium of slowly interconverting on the NMR time scale folded and unfolded conformations. Two sets of resonances originating from folded and unfolded conformations are observed in the [^1^H,^15^N]-HSQC spectrum of the PAI subdomain at pH 5.0 in the presence of 250 mM NaCl (Figure 4A). This property of the PAI subdomain provides a unique opportunity to monitor the binding of DNA to each conformation independently. Here, we investigated the ability of folded and unfolded PAI subdomain to bind the 18 bp DR-core sequence, that represents the minimal sequence required for transposase binding [35].

Previously, we have shown that the DNA-binding of the PAI subdomain occurs in the intermediate regime on the NMR time scale leading to the broadening of the PAI resonances caused by exchange between the DNA-bound and unbound states [7]. We therefore analyzed the effects of the protein binding to the transposon DNA on the basis of retention of peak intensities for each residue. To determine whether the transposon DNA binds to the folded, unfolded, or both conformations of the PAI subdomain, we monitored the changes of resonance intensities in the [^1^H,^15^N]-HSQC spectra upon the addition of increasing to saturation concentrations of DR-core DNA. A set of non-overlapping resonances observed for both PAI conformations was used for the analysis. This set comprised the resonances originating from Q11, D12, V18, G28, A29, R38, Y48, and H50 amino acid residues (Figure 4 A). Of these residues, G28, G29, and R38 were located in the DNA-binding site (Figure 4 B). While the folded conformation of the PAI subdomain demonstrates well-dispersed resonances in the [^1^H,^15^N]-HSQC spectrum, the number of residues that can be used in the analysis was limited due to overlap of many resonances originated from the unfolded PAI subdomain. Figure 4C shows resonance intensities as the function of increasing to saturation concentrations of the DNA-core, normalized by the intensity of the respective resonance measured in its absence. Notably, only the resonances corresponding to the folded PAI subdomain appear to be affected by the presence of DNA-core sequence, indicating that it is the folded PAI subdomain that binds to the transposon DNA. As expected, the resonances originating from the residues in the DNA-binding site show the largest changes.

## Discussion

DNA transposition requires the formation of higher-order nucleoprotein complex and begins with the binding of the transposase enzyme to the transposon DNA. Macromolecular interactions, including protein-DNA interactions, depend on the three-dimensional structures of both interacting partners. Here, we show that the folding of the primary DNA-recognition subdomain of SB transposase, i.e. the PAI subdomain, depends on environmental conditions, in particular on temperature. It appears that the PAI subdomain exists as a temperature-dependent ensemble of interconverting folded and unfolded confirmations. Based on [^1^H,^15^N]-HSQC NMR and intrinsic tyrosine fluorescence analysis, at pH 7.0 (e.g., close to physiologic pH), the presence of unfolded conformation becomes increasingly significant as the temperature increases above 15°C (Figures 1 and 2). Furthermore, we show that the transposon DNA preferentially binds to the folded conformation of the PAI subdomain, suggesting that whether the PAI subdomain is folded or unfolded *in situ* may affect the DNA-binding properties of SB transposase. In this regard, our results imply that if we consider only the folding properties of the protein, the choice of temperature in designing transposition experiments could be important for optimal SB transposase activity.

The results presented here, also provide an insight into the mechanism of coupled folding and DNA-binding of the PAI subdomain. The exchange rate between folded and unfolded conformations of the PAI subdomain is slow on the NMR time scale allowing us to observe the binding of DNA to each conformation separately. We have determined that the PAI subdomain must be folded before binding to DNA, suggestive of the “conformational selection” model of molecular recognition [36]–[38]. However, the process of DNA-binding by the PAI subdomain is more complex and involves subsequent conformational adaptation. First, this is evident from the observation of decreasing resonance intensities upon the addition of DNA for residues Q11, D12, V18, G28, A29 located away from the DNA-binding site of the PAI subdomain (Figure 4B). The fact that these residues are affected by the presence of DNA suggests that they are involved in the conformational rearrangement caused by the binding of the PAI subdomain to the DNA-core sequence. Next, the orientation of helix 2, which is the part of the helix-turn-helix motif that binds to DNA, is significantly different in the DNA-free state of the PAI subdomain and DNA-bound states of respective subdomains in related transposases Tc3 and Mos1 [7]. It is likely that the transposon DNA is first recognized by the pre-folded PAI subdomain via conformational selection and, subsequently, the PAI subdomain undergoes structural reorganization involving the re-orientation of helix 2, reminiscent of the “induced fit” model [39]. This scenario is in agreement with a general understanding that the conformational selection and the induced fit models are two extreme mechanistic possibilities, but in real systems conformational selection is often followed by conformational adjustment [37], [40] as has been shown in the number of cases [41]–[43]. Our model of the PAI subdomain binding to DNA is also in agreement with the finding that strong and long range protein-ligand interactions favor the induced fit model, while weak and short range interactions favor the conformational selection model [44]. Indeed, the fact that the binding of the PAI subdomain to DNA occurs in the intermediate regime on the NMR time scale, indicates that the PAI:DNA interactions are relatively weak.

The SB transposon is a widely used tool in genetic applications. Moreover, currently it is in the first in-human clinical trial for treatment of patients with B-lymphoid malignancies [4], [45]. In attempt to have a better controlled gene integration, direct fusions of target-specific DNA-binding domains to SB transposase were produced [46]–[47]. However, such fusions markedly decreased or diminished the SB transposase activity [48]. The hyperactive version of SB transposase, SB100X, remained active as a fusion transposase; however, precise genomic site-directed integration has not been achieved [49]. The results of our study show that depending on the environmental conditions the fraction of the unfolded PAI subdomain may become significant, yet the folding of the PAI subdomain is needed for the SB transposase to bind the transposon DNA. We speculate that one of the reasons for the limited success of SB transposase fusions to the target-specific DNA-binding domains could be that the added sequences compromised the folding of the PAI subdomain.

In summary, the presented analysis reveals the mechanism of DNA recognition by the primary DNA-recognition subdomain of SB transposase and represents a step toward a molecular-level understanding of the complex pathways involved in SB transposition. On a practical note, increasing the structural stability of the PAI subdomain could be beneficial for the activity of SB transposase.
